# Supplementary material for: Long COVID and Hybrid Immunity among Children and Adolescents Post-Delta Variant Infection in Thailand
Source: Vaccines (Basel). 2023 Apr 23;11(5):884. doi: 10.3390/vaccines11050884 (PMC10223729; doi:10.3390/vaccines11050884)
Supplement: Supplementary file 1 [file vaccines-11-00884-s001.zip › vaccines-2294046-supplementary.pdf]

The study of post-COVID-19 conditions and follow immunogenicity among children and adolescents post-SARS-CoV-2 infection in King Chulalongkorn Memorial Hospital

Visit 1

Questionnaires for children and adolescents Subject No.| 4 | 1 |__|__|__|

Initial |__|__|

The questionnaires of health problems after SARS-CoV-2 infection

**Demographic and baseline clinical characteristics**
Date to collect data<br>(Day/month/year) ☐☐/☐☐/☐☐☐☐<br>1. Gender O Female O Male<br>2. Aged _________ year _________ month _________ day<br>3. Height _________ cm<br>4. Bodyweight _________ kg<br>5. Date of diagnosis SARS-CoV-2 infection ________________________<br>6. Diagnosis O Asymptomatic O Symptomatic<br>7. Definite diagnosis. O Upper respiratory tract infection O Pneumonia<br>O Other, describe; _______________________<br>8. Underlying disease O No O Underlying disease, describe; _______________________<br>9. Place in treatment when diagnosis of SARS-CoV-2 infection<br>O Ward O ICU O Hospitel O Community isolation O Home isolation<br>10. Respiratory support when infected<br>O Oxygen canula or mask O HHHFNC O Non-invasive PPV O Mechanical ventilation<br>11. mRNA (BNT162b2) vaccination<br>O 1 dose O 2 doses O More than 2 doses<br>O Date of received first dose of COVID-19 vaccine ☐☐/☐☐/☐☐☐☐<br>O Date of received second dose of COVID-19 vaccine ☐☐/☐☐/☐☐☐☐<br>O Date of received other dose of COVID-19 vaccine _______________________<br><br>Sign ____________________________ Date _______________

**1. Residual or new symptoms after recovery from acute SARS-CoV-2 infection past 14 days to 3 months before this visit**
O No O Yes, please check mark in this below

1. Fever	O No	O Yes	O N/A
2. Fatigue	O No	O Yes	O N/A
3. Muscle pain	O No	O Yes	O N/A
4. Headache	O No	O Yes	O N/A
5. Cough	O No	O Yes	O N/A
6. Sore throat	O No	O Yes	O N/A
7. Anosmia	O No	O Yes	O N/A
8. Rhinorrhea/nasal congestion	O No	O Yes	O N/A
9. Chest pain or chest tightness	O No	O Yes	O N/A
10. Dyspnea	O No	O Yes	O N/A
11. Difficulty breathing	O No	O Yes	O N/A
12. Rash	O No	O Yes	O N/A
13. Insomnia	O No	O Yes	O N/A
14. Diarrhea	O No	O Yes	O N/A
15. Constipation	O No	O Yes	O N/A
16. Abdominal pain	O No	O Yes	O N/A
17. Hair loss	O No	O Yes	O N/A
18. Abnormal movement	O No	O Yes	O N/A
19. Abnormal posture	O No	O Yes	O N/A
20. Somnolence	O No	O Yes	O N/A
21. Weight loss	O No	O Yes	O N/A
22. Difficulty swallowing	O No	O Yes	O N/A
23. Loss of appetite	O No	O Yes	O N/A
24. Arthralgia	O No	O Yes	O N/A
25. Dizziness	O No	O Yes	O N/A
26. Syncope	O No	O Yes	O N/A
27. Blurred vision	O No	O Yes	O N/A
28. Urinary problems	O No	O Yes	O N/A
29. Loss of taste	O No	O Yes	O N/A
30. Tremor	O No	O Yes	O N/A
31. Numbness	O No	O Yes	O N/A
32. Loss of concentration	O No	O Yes	O N/A
33. Irregular menstruation	O No	O Yes	O N/A
34. Conjunctivitis	O No	O Yes	O N/A
35. Communication skill problems	O No	O Yes	O N/A
36. Seizure	O No	O Yes	O N/A

37. Other symptoms, if any, please describe; ___________________________

38. Summary of symptoms; ______________ symptom

Sign _____________________________ Date _______________

**2. Diagnosis of complications after SARS-CoV-2 infection**
O No<br>O multisystem inflammatory syndrome Pulmonary embolism /micro emboli (PE, "Clot in lung")<br>O Kawasaki disease<br>O Multisystem inflammatory syndrome (MIS-C/PIMS-TS)<br>O Respiratory failure<br>O Asthma<br>O Myocarditis (inflammation of the heart muscle)<br>O Depression<br>O Anxiety<br>O Diabetes O Type 1 O Type 2<br>O Shock / Toxic shock syndrome<br>O Coagulopathy (excessive bleeding or clotting)<br>O Kidney problems<br>O Intussusception<br>O SARS-CoV-2 re-infection, date of re-infection____________________<br>Other diagnosis, please describe; __________________________________<br><br>Sign ____________________________ Date _______________

3. Physical examination: BT ☐☐.☐ C, HR ☐ bpm, BP☐☐/☐ mmHg, RR☐ /min

General appearance	ONormal	OAbnormal_____________________	OSignificant	Onot Significant
Skin	ONormal	OAbnormal_____________________	OSignificant	O not significant
HEENT	ONormal	OAbnormal_____________________	OSignificant	O not significant
Cardiovascular system	ONormal	OAbnormal_____________________	OSignificant	O not significant
Pulmonary system	ONormal	OAbnormal_____________________	OSignificant	O not significant
Abdomen	ONormal	OAbnormal_____________________	OSignificant	O not significant
Lymphatic system	ONormal	OAbnormal_____________________	OSignificant	O not significant
Other	ONormal	OAbnormal_____________________	OSignificant	O not significant

Comments:____

Sign_________________________________ Date___________________

4. Investigations

CBC O Hb___________
AST__________ U/L ALT_________________ U/L
Neutralizing antibody against Delta variants ____________________ % inhibition
Neutralizing antibody against Omicron variants ____________________ % inhibition
anti-S-RBD IgG _________________ BAU/mL
Film chest x-ray O 0. Normal O 1. Abnormal
If Abnormal chest x-ray O 1. bilateral or unilateral ground glass opacities
O 2. bilateral of unilateral interstitial infiltration
O 3. bilateral or unilateral consolidation
O 4. bilateral of unilateral pleural effusion

Sign_________________________________ Date___________________
